# The Lipoxygenase Lox1 Is Involved in Light‐ and Injury-Response, Conidiation, and Volatile Organic Compound Biosynthesis in the Mycoparasitic Fungus *Trichoderma atroviride*

**DOI:** 10.3389/fmicb.2020.02004

**Published:** 2020-08-27

**Authors:** Verena Speckbacher, Veronika Ruzsanyi, Ainhoa Martinez-Medina, Wolfgang Hinterdobler, Maria Doppler, Ulrike Schreiner, Stefan Böhmdorfer, Marzia Beccaccioli, Rainer Schuhmacher, Massimo Reverberi, Monika Schmoll, Susanne Zeilinger

**Affiliations:** ^1^Department of Microbiology, University of Innsbruck, Innsbruck, Austria; ^2^Institute for Breath Research, University of Innsbruck, Innsbruck, Austria; ^3^Plant-Microbe Interaction Unit, Institute of Natural Resources and Agrobiology of Salamanca (IRNASA-CSIC), Salamanca, Spain; ^4^Center for Health and Bioresources, AIT Austrian Institute of Technology, Tulln, Austria; ^5^Institute of Bioanalytics and Agro-Metabolomics, Department of Agrobiotechnology (IFA-Tulln), University of Natural Resources and Life Sciences, Vienna (BOKU), Tulln, Austria; ^6^Department of Chemistry, University of Natural Resources and Life Sciences (BOKU), Tulln, Austria; ^7^Department of Environmental Biology, Sapienza University, Rome, Italy

**Keywords:** 6-pentyl-α-pyrone, injury, filamentous fungus, ascomycetes, secondary metabolites, volatile organic compound, mycoparasitism, oxylipins

## Abstract

The necrotrophic mycoparasite *Trichoderma atroviride* is a biological pest control agent frequently applied in agriculture for the protection of plants against fungal phytopathogens. One of the main secondary metabolites produced by this fungus is 6-pentyl-α-pyrone (6-PP). 6-PP is an organic compound with antifungal and plant growth-promoting activities, whose biosynthesis was previously proposed to involve a lipoxygenase (Lox). In this study, we investigated the role of the single lipoxygenase-encoding gene *lox1* encoded in the *T. atroviride* genome by targeted gene deletion. We found that light inhibits 6-PP biosynthesis but *lox1* is dispensable for 6-PP production as well as for the ability of *T. atroviride* to parasitize and antagonize host fungi. However, we found Lox1 to be involved in *T. atroviride* conidiation in darkness, in injury-response, in the production of several metabolites, including oxylipins and volatile organic compounds, as well as in the induction of systemic resistance against the plant-pathogenic fungus *Botrytis cinerea* in *Arabidopsis thaliana* plants. Our findings give novel insights into the roles of a fungal Ile-group lipoxygenase and expand the understanding of a light-dependent role of these enzymes.

## Introduction

*Trichoderma atroviride* is a filamentous ascomycete exhibiting a necrotrophic mycoparasitic lifestyle with high antagonistic activity against a broad range of plant pathogenic fungi (Karlsson et al., 2017). *T. atroviride* in addition is capable of priming the plant’s immune system, thereby inducing systemic resistance as well as enhancing the plant’s resilience against adverse environmental impacts, and promoting plant growth (Harman et al., 2004; Mukherjee et al., 2013b; Lee et al., 2015). All these beneficial properties result in the frequent application of *T. atroviride* in agriculture as a biological control agent against various fungal plant diseases (Verma et al., 2007; Mukherjee et al., 2013a).

Secondary metabolites – which are not primarily necessary for growth and survival of an organism but favor the perseverance and ecologic success – are among the main agents determining the strength and progress of the mycoparasitic attack (Kubicek et al., 2011; Mukherjee et al., 2012). Depending on the environmental conditions, *T. atroviride* produces a plethora of secondary metabolites (e.g., non-ribosomal peptides, polyketides, and terpenoids; Hermosa et al., 2014; Zeilinger et al., 2016). These include volatile organic compounds (VOCs; Stoppacher et al., 2010), which influence the mycoparasitic activity, as well as the interaction of *T. atroviride* with plants (Druzhinina et al., 2011; Lee et al., 2015; Holzlechner et al., 2016). One of the main secondary metabolites of *T. atroviride* is 6-pentyl-α-pyrone (6-PP), an unsaturated lactone derived from fatty acid metabolism that exhibits antifungal activity and concentration-dependent plant-growth promoting characteristics (Vinale et al., 2008). Despite the biocontrol-associated activities of 6-PP, the pathway underlying its biosynthesis still awaits clarification. Based on isotopic labeling experiments the oxidation of linoleic acid to 13S-hydroperoxy-9Z,11E-octadecadienoic acid (13-HPOD) by a lipoxygenase (Lox) was proposed as the first and limiting step in the biosynthesis of 6-PP by *T. atroviride* (Serrano-Carreon et al., 1993). Accordingly, 6-PP producing *Trichoderma* species like *T. atroviride*, *Trichoderma gamsii* and *Trichoderma harzianum* encode a *lox* gene in their genomes, while non-producers such as *Trichoderma virens* and *Trichoderma reesei* do not (Atanasova et al., 2013; Zeilinger et al., 2016).

Lipoxygenases are non-heme iron‐ or manganese-containing dioxygenases catalyzing the oxidative conversion of polyunsaturated fatty acids to fatty acid hydroperoxides (therefore also designated as oxylipins). C18 polyunsaturated fatty acids, such as linoleic and α-linolenic acid, are the predominant lipoxygenase substrates in plants and fungi (Andreou et al., 2009). Two lipoxygenase groups exist in fungi: Val-group lipoxygenases bear a C-terminal valine and contain a conserved WL-L/F-AK motif, which is also characteristic for lipoxygenases from plants and animals (Heshof et al., 2014). Ile-group lipoxygenases have a C-terminal isoleucine and contain a conserved WRYAK motif, which is characteristic for fungi (Heshof et al., 2014). The numerous roles of lipoxygenases and their products are well investigated in plants and animals, while knowledge on these enzymes is lacking behind in filamentous fungi (Andreou et al., 2009). The Ile-group lipoxygenase LoxB was reported to be involved in spore swelling and programmed spore germination in *Aspergillus fumigatus* (Fischer et al., 2017). Deletion of the *Aflox* gene resulted in a diminished quorum-sensing dependence of the balancing between conidia and sclerotia formation in *Aspergillus flavus* (Horowitz Brown et al., 2008). Recently, the deletion of the two Ile-group lipoxygenase-encoding genes in *Podospora anserina* was reported to eradicate the production of certain C7-VOCs, thereby leading to a decreased ability of the fungus to repel the nematode *Caenorhabditis elegans* (Ferrari et al., 2018).

This study was initiated with the aim to generate *T. atroviride* mutants defective in the production of 6-PP by deleting the *lox1* gene, based on the isotopic labeling experiments of Serrano-Carreon et al. (1993). Here, we show that 6-PP biosynthesis is governed by light. However, against expectations, *lox1* was dispensable for the biosynthesis of 6-PP in *T. atroviride* and respective ∆*lox1* gene deletion mutants had mycoparasitic activities similar to the wild-type. Further characterization of the ∆*lox1* mutants revealed that Lox1 was involved in asexual sporulation in darkness, including the conidiation response of *T. atroviride* to injury. In addition, the production of several metabolites such as the VOCs 1-octen-3-ol, 2-heptanone, and 3-octanone was Lox1 dependent in darkness and the deletion of *lox1* led to an enhanced priming of the *Arabidopsis thaliana* immune response against *Botrytis cinerea*.

## Materials and Methods

### Strains and Cultivation Conditions

*T. atroviride* P1 (ATCC 74058; Ascomycota), its mutant strains ∆*tga1* (Reithner et al., 2005), ∆*tmk1* (Reithner et al., 2007) and *gpr1*-sil (Omann et al., 2012), and the host fungi *Rhizoctonia solani* (Basidiomycota; pathogenic isolate obtained from the collection of the Institute of Plant Pathology, Università degli Studi di Napoli “Federico II,” Naples, Italy), *B. cinerea* B05.10 (Ascomycota) and *Fusarium oxysporum* f. sp. *lycopersici* strain 4287 (Ascomycota) were used throughout this study. For pre-cultivation, wild-type strains were pre-grown for 2 days on potato dextrose agar (PDA; Becton, Dickinson and Company, Le Pont De Claix, France) plates and mutant strains on PDA plates supplemented with 200 μg/ml hygromycin B (Calbiochem®, Merck KGaA, Darmstadt, Germany). An agar plug (5 mm) of the actively growing colony margin was then propagated twice, after 2 days each, to the center of a fresh PDA plate, to reach exponential growth under light-dark (12:12 h cycle; 300 Lux; Snijders Micro Clima-Series TM Labs Economic Lux Chamber; Snijders Labs, Tiburg, Netherlands) conditions or in complete darkness. Plates were incubated in an upright position without parafilm.

For determination of the radial growth rate on solid media, an agar plug (5 mm) of the actively growing colony margin of the final pre-culture was transferred in three biological replicates to the center of fresh PDA plates. The fungi were grown at 28°C under light-dark conditions. The colony radii were measured after 30 and 55 h and the radial growth rate (cm/d) was calculated for each time point. Statistical analysis and plots were done in Biovinci 1.1.5.

Biomass production, conidial germination and germ tube development were assessed in at least three biological replicates by inoculating 1 × 10^6^ spores/ml medium in 50 ml of potato dextrose broth (PDB; Becton, Dickinson and Company, Le Pont De Claix, France) in 125 ml Erlenmeyer flasks. Cultures were incubated at 28°C and 250 rpm. Mycelial dry weight (DW) was determined after 50 h incubation to assess biomass production (g DW/l). Conidial germination rate and germ tube length were determined with a Thoma chamber (0.1 mm depth, 0.0025 mm^2^, Profondeur; Brand GmbH + Co KG, Wertheim, Germany), a Nikon optiphot-2 microscope and Nikon NIS Elements D Software after 5.5, 7, 8, 9, 10, 11, and 13 h of incubation. Statistical analysis and plots were done in Biovinci 1.1.5.

### Bioinformatics Analysis and Generation of Deletion Mutants

Lox1 (Ta_33350)^1^ was verified as a potential lipoxygenase by protein domain prediction using the Prosite database (de Castro et al., 2006). For generation of *lox1* deletion mutants, a 1,010 bp fragment spanning the 5' non-coding region (amplified with primers 33350-5KO-F1 and 33350-5KO-R1; Supplementary Table 1) and a 1,009 bp fragment spanning the 3' non-coding region (amplified with primers 33350-3KO-F1 and 33350-3KO-R1; Supplementary Table 1) of the *lox1* gene, as well as the hygromycin-resistance conferring selection-marker (*hph*) cassette (Hartl et al., 2007) were combined by yeast recombinational cloning (Oldenburg et al., 1997; Colot et al., 2006; Schuster et al., 2012). Transformation of *T. atroviride* protoplasts was performed as described previously (Gruber et al., 1990). Resulting transformants were selected on PDA containing 200 μg/ml hygromycin B (Calbiochem®, Merck KGaA, Darmstadt, Germany) and purified to mitotic stability by three rounds of single spore isolation.

Deletion of *lox1* and locus specific integration of the hygromycin resistance cassette was verified by PCR using gene‐ and locus-specific primer pairs. Primers 33350-RT-F1 and 33350-C-R1 (Supplementary Table 1) bind inside the open reading frame of the *lox1* gene and outside of the sequence covered by the deletion cassette; primers 33350-C-F1 and hph-C-R1-right (Supplementary Table 1) bind inside the open reading frame of the hygromycin resistance cassette and outside of the sequence covered by the deletion cassette. Absence of *lox1* messenger RNA (mRNA) was confirmed by RT-qPCR with primers 33350-RT-F1 and 33350-RT-R1 (primer efficiency of 99% at 300 nM primer concentration and 62°C annealing temperature). Correct integration and copy number determination of the deletion cassette was confirmed by Southern blot analysis. High-integrity genomic DNA was digested with *Bsa*I and *Bgl*II yielding a fragment of 5,797 bp that spans from the 5' to the 3' non-coding region outside of the homologous recombination sites of the *hph*-cassette. A DIG labeled probe amplified with primers hph-F2 and hph-R and binding within the *hph* gene was used for hybridization (Supplementary Table 1).

### Transcriptional Analysis by RT-qPCR

For transcript analysis, agar plugs from the actively growing colony margin were transferred to the center of fresh PDA plates covered with a cellophane membrane and incubated at 25°C under light-dark conditions. Total RNA from the harvested, actively-growing colony margin was extracted using TRIzol Reagent (Invitrogen, Karlsruhe, Germany) as described previously (Gruber and Zeilinger, 2014). Isolated RNA was treated with DNAse I and reverse transcribed with the RevertAid H Minus First Strand complementary DNA (cDNA) Synthesis Kit (ThermoFisher Scientific Baltic UAB, Vilnius, Lithuania) with a 1:1 combination of the provided oligo(dT) and random hexamer primers. qPCR was performed with GoTaq qPCR Master Mix (Promega Corporation, Madison, USA) and a qTOWER^3^ G cycler (Analytik Jena AG, Jena, Germany) with the primers 33350-RT-F1 and 33350-RT-R1 (Supplementary Table 1). Expression ratios were calculated according to Pfaffl (2001) by normalizing to the basal expression levels of *T. atroviride* wild-type growing alone and by using *sar1* as reference gene (Brunner et al., 2008). Three biological replicates were pooled as one sample. Three pooled samples were analyzed in at least three technical replicates. Data analysis was done using qPCRsoft 4.0 software (Analytik Jena AG, Jena, Germany). Statistical analysis and plots were done in Biovinci 1.1.5.

### Dual Confrontation Assays

Dual confrontation assays of *T. atroviride* versus the host fungi *R. solani*, *B. cinerea*, and *F. oxysporum* were set up in four biological replicates as described previously (Lorito et al., 1996; Zeilinger et al., 1999). Plates were incubated at 25°C under light-dark conditions or constant darkness for 6 days. Pictures of light-dark incubation were captured after 4 and 6 days; in case of incubation in complete darkness pictures were taken after 6 days at the end of the incubation phase. Image editing was done in GIMP 2.10.20.

### Inhibition Assay and Quantification of Secreted 6-PP and Oxylipins

The inhibitory effect of *T. atroviride*-secreted, diffusible metabolites on *B. cinerea* spore germination and growth was determined according to Graeme-Cook and Faull (1991) with slight modifications. PDA plates covered with a cellophane or dialysis membrane (MWCO 14000; Carl Roth GmbH + Co. KG, Karlsruhe, Germany) were inoculated in four biological replicates and incubated at 28°C for 33 h in darkness. After marking the margins of the *Trichoderma* colonies, the membrane was removed and each plate was inoculated with 1.62 × 10^6^ *B. cinerea* spores, followed by incubation at room temperature for 68 h. Inhibition zones were measured, and the inhibition index was calculated as a quotient of the diameter of the inhibition zone of *B. cinerea* and the diameter of the former colony margin of *T. atroviride*.

For determination of secreted 6-PP, *T. atroviride* was cultivated for 48 h at 25°C either in darkness or under light-dark conditions on cellophane-covered PDA plates in four biological replicates. After removal of the *Trichoderma*-covered membrane, 1 g of agar situated below the colony was harvested. 5 ml of the extraction solvent, consisting of methanol (MeOH):H_2_O 3:1 (v/v) + 0.1% formic acid (FA), were added to 1 g of agar (MeOH: Merck, Darmstadt, Germany; water purified with ELGA Purelab Ultra-AN-MK2: Veolia Water, Vienna, Austria; FA: MS-grade, Sigma-Aldrich, Vienna, Austria) and sonicated for 15 min. Acidified water (0.5 ml) containing 0.1% FA was added to 1 ml extract to obtain an organic-solvent:water ratio of 1:1 (v/v). For liquid-chromatography coupled to high-resolution mass spectrometry (LC-HRMS) analysis, the samples were additionally diluted 1:10 (v/v) with MeOH:H_2_O 1:1 (v/v) + 1% FA. Samples were analyzed on an LC-HRMS system consisting of a Vanquish ultra-high-performance liquid chromatography (UHPLC) coupled to a QExactive Orbitrap HF mass spectrometer (Thermo Fisher Scientific, Bremen, Germany). Two microliters of the sample were injected and chromatographed on a reversed phase C18 column XBridge 150 × 2.1 mm i.d., 3.5 μm (Waters, Milford, USA). H_2_O and MeOH, both with 0.1% FA, were used as eluents A and B, respectively, to obtain a linear-gradient elution with increasing MeOH content. After an initial hold time of 1 min at 10% eluent B, the methanol content was increased to 100% within 9 min (3 min hold) before the system was re-equilibrated for 7 min at 10% eluent B (total run time 20 min). Flow rate was kept constant at 0.25 ml/min. Mass spectra were recorded from *m/z* 100 to 1,000 in positive ionization mode with a resolving-power setting of 120,000 at *m/z* 200. Quantification was carried out using the XCalibur software (Thermo Fisher Scientific, Bremen, Germany) after external standard calibration of 6-PP (purity > 96%; 1, 5, 10, 50, 100, 500, 1,000, and 5,000 μg/L; Sigma-Aldrich, Vienna, Austria). Values were normalized to the mycelial dry weight. Statistical analysis and plots were done in Biovinci 1.1.5.

Oxylipins were extracted as previously reported (Ludovici et al., 2014) from 30 mg of lyophilized biomass obtained from plate cultures incubated as described above. Briefly, samples were extracted with 2 ml of 1:1:3 isopropanol:water:ethyl acetate (v/v) in presence of 0.0025% butylated hydroxytoluene (w/v) to prevent peroxidation. In each sample, the internal reference standard 9(S)-HODE-d_4_ (Cayman Chemical) was added at the concentration of 1 μM, calculated on the final resuspension volume of 100 μl. Samples were vortexed for 5 min, centrifuged at 12,000 rpm, and the upper phase collected and dried with nitrogen gas. Each sample was extracted a second time with 1 ml of ethyl acetate. After centrifugation at 12,000 rpm, the upper phase was transferred into a collection tube with the first extract and dried with nitrogen gas. The combined extracts were dissolved in 100 μl of MeOH and analyzed by LC-MS/MS. Chromatographic separation of oxylipins was performed with a Zorbax SB-C8 rapid resolution column (HT 2.1 × 50 mm 1.8 μm 600 bar, Agilent Technologies). The mobile phases consisted in phase A of 97:3 water:acetonitrile (v/v in 0.1% of formic acid) and in phase B of 90:10 acetonitrile:isopropanol (v/v). The elution program was: 0–2 min 20% B, 2–4 min 35% B, 4–6 min 40% B, 6–7 min 42% B, 7–9 min 48% B, 9–15 min 65% B, 15–17 min 75% B, 17–18.5 min 85% B, 18.5–19.5 min 95% B, 19.50–24 min 95% B, 24–26 min 99% B, 26–30 min 99% B, 30–34 min 20% B. The flow rate was: 0–24 min 0.6 ml/min, 24–30 min 1 ml/min, 30–34 min 0.6 ml/min. The column was heated to 50°C with an injection volume of 10 μl. Oxylipins were identified by multiple reaction monitoring (MRM) approach (Ludovici et al., 2014; Supplementary Table 2). Peak areas were normalized to the internal reference standard. Statistical analysis and plots were done in Biovinci 1.1.5.

### Mechanical Injury and Blue-Light-Induced Conidiation Assays

Mechanical injury and blue-light-induced conidiation assays were done in at least four biological replicates according to Hernández-Oñate et al. (2012) with slight modifications. PDA plates supplemented with 4 mg/ml ascorbic acid were inoculated with agar plugs (5 mm) from pre-cultures and incubated at 25°C. To assess the effect of light, the plates were incubated in an upright position under either light-dark conditions or in complete darkness for 5 days. Alternatively, a blue-light-pulse was set to cultures grown under complete darkness for 48 h by illuminating the plates for 10 min with a blue-light-source (300–400 nm, 25 Lux, distance 19 cm; Blacklight Blue UV-A Lamp Supratec L18 W/73; Osram GmbH, Garching, Germany), followed by incubation under complete darkness for a further 72 h. To assess induction of conidiation by injury, mycelial injury was set after 48 h of growth under complete darkness by cutting a grid pattern with a scalpel under red safety light. Further incubation was done under complete darkness for 72 h. Conidia were harvested and counted with a Thoma chamber (0.1 mm depth, 0.0025 mm^2^, Profondeur; Brand GmbH + Co KG, Wertheim, Germany). Statistical analysis and plots were done in Biovinci 1.1.5. Image editing was done in GIMP 2.10.20.

For high performance thin-layer chromatography (HPTLC) analysis, three biological replicates – each consisting of three 1.2 cm agar discs including mycelia from one plate (in total 3.4 cm^2^ and approximately 1.4 g) – were harvested per sample to a 15 ml centrifuge tube and frozen in liquid nitrogen. Three independent biological replicates were analyzed for each strain and condition.

### Metabolite Extraction and High Performance Thin-Layer Chromatography

HPTLC analysis was done according to Hinterdobler et al. (2019) with some modifications. Metabolites were extracted from the agar by supersonication for 15 min in a mixture of 5 ml water (p.A.) and 4 ml ethyl acetate (EtOAc). Liquid-liquid extraction with 4 ml EtOAc was repeated two times. The collected organic phase was evaporated, re-collected in 140 μl methanol (MeOH) and 8 μl were applied to HPTLC separation and analysis. Samples were spotted on a silica gel plate (HPTLC silica gel 60 F_254_S, Merck KGaA, Darmstadt, Germany) and separated with chloroform:1 mM trifluoroacetic acid in MeOH 7:1 (v/v) as mobile phase. Pictures were taken at different wavelengths before and after derivatization with p-anisaldehyde:sulfuric acid reagent.

### Determination of VOC Production

For determination of VOC production, 150 ml Schott bottles filled with 25 ml of PDA were inoculated at the outmost margin with a mycelia-covered agar plug (5 mm) and closed with Teflon® screw-caps (Bohlender™, Merck, Vienna, Austria) with two openings for air in and outlet. The cultures were pre-grown for 20 h at 25°C, under either light-dark conditions or complete darkness in four biological replicates. After 20 h of cultivation, the four samples in Schott bottles were held in an incubator adapted for headspace measurements (Supplementary Figure 1) and connected in a gastight way, parallel to the purified air supply and to the sampling outlet. To avoid condensation, the temperatures of the water bath and headspace air were 23 ± 2 and 40°C, respectively. Purified air with 5 ml/min flow was applied, streaming through every flask continuously, regulated by mass flow controllers (Bronkhorst, Ruurlo, Netherlands) integrated into the incubator. Additionally, 5 ml/min dilution flow was connected after the flasks to reduce moisture in the samples. Samples were collected in the incubator at 40°C in 100 and 250 ml glass syringes (Socorex, Ecublens, Switzerland). Photos of light-dark samples were taken at each sampling time point to document progress of conidiation and of samples from complete darkness solely at the end of the experiment to avoid exposure to light.

### Gas Chromatography – Ion Mobility Spectrometry Analysis of VOCs

The produced VOCs were monitored using an in-house made high resolution gas chromatography – ion mobility spectrometry (GC-IMS) developed at Leibniz University, Hannover, Germany. Hundred-milliliter glass syringes were connected immediately after sampling to the heated inlet (40°C) of the GC-IMS. Samples were injected into the GC column using a stainless steel sample loop (200 μl) installed on a six-way valve. Volatiles were separated using a RTX volatiles column (10 m × 0.53 mm, film thickness 2 μm; Restek Corporation, Bellefonte, USA) working at constant temperature of 50°C. The carrier-gas flow-rate program was as follows: 3 ml/min for 10 min and then 10 ml/min for another 10 min, resulting in a total GC-runtime of 20 min. The IMS, with a drift tube length of 7.5 cm, provided a resolving power of *R* = 90 using a drift voltage of 5 kV. The instrument operated at 40°C, 10 mbar above the ambient pressure and with the purified air as the drift gas at the flow of 150 ml/min. A radioactive β^−^emitter ^3^H (300 MBq) was used as ionization source. A detailed description of the system can be found elsewhere (Langejuergen et al., 2015). Quantification was carried out using LAV software (Version 2.2.1, GAS mbH, Dortmund, Germany) after external standard calibration of 1-octen-3-ol, 2-heptanone, and 3-octanone (purchased from Sigma Aldrich, purity higher than 98%, respectively). Test gases of the compounds were prepared in the concentration range of 1–200 ppb as described elsewhere (Mochalski et al., 2018). Chromatographic data were acquired using Agilent Chemstation Software (GC-MS Data Analysis, Agilent, Waldbronn, Germany) and the mass spectrum library NIST 2008 (Gatesburg, USA) was applied for identification. Statistical analysis and plots were done in Biovinci 1.1.5.

### *A. thaliana* Growth-Promotion and Pathogen-Resistance Bioassay Against *B. cinerea*

Surface sterilized *A. thaliana* (wild-type accession Col-0) seeds were sown in one compartment of two-compartment circular plates (120 mm diameter) on Murashige and Skoog agar-solidified medium (pH 6) supplemented with 0.5% sucrose and 2.5 mM 2-(*N*-morpholino)ethanesulfonic acid (MES) according to Martínez-Medina et al. (2017). After 2 days of stratification at 4°C, the plates were positioned vertically and incubated at 22°C in a 10 h:14 h light-dark cycle (light intensity 100 μmol/m^2^/s) for 12 days.

As a positive control for induction of systemic resistance, *T. harzianum* T-78 (CECT 20714 of the Spanish Type Culture Collection, Valencia, Spain; Martínez-Medina et al., 2013) was used and compared with *T. atroviride* wild-type and ∆*lox1* mutants. The fungal strains were pre-cultured at 28°C on PDA plates for 5 days. An agar-plug (7 mm) from the actively growing colony margin was transferred into the plant-free compartment of the two-compartment circular plates. The plates were sealed with one layer of gas-permeable Parafilm® and placed in a vertical position in the growth chamber. The exchange of VOCs in-between the seedlings and the *Trichoderma* cultures was allowed for pathogen-resistance bioassays for 3 days, and for growth-promotion assays for 6 days.

Growth-promotion was assessed by determining the average plants shoot fresh-weight after 6 days of VOC treatment. Furthermore, the average total length of lateral roots, as well as the average number of lateral roots was measured with ImageJ. The experiment was performed in triplicates with 10 plants per plate. One-way ANOVA was applied with SPSS Statistics V25 for windows (Tukey HSD test; *p* < 0.05, *n* = 3 plates/30 plants).

For the pathogen-resistance bioassay, 15 days old *A. thaliana* seedlings, which were VOC treated for 3 days, were transferred to 60 ml pots containing a sterile 5:12 sand:soil (v/v) mixture. Pots were incubated in a growth chamber in an 8 h (24°C):16 h (20°C) light-dark cycle (light intensity 100 μmol/m^2^/s) at 70% relative humidity for 3 weeks according to Martínez-Medina et al. (2017). Plants were watered every day and received half-strength Hoagland solution containing 10 μM Sequestrene (CIBA-Geigy AG, Basel, Switzerland) once a week. After 3 weeks, the VOC-treated *A. thaliana* plants were inoculated with *B. cinerea* strain B05.10 (van Kan et al., 1997) according to van Wees et al. (2013) with slight modifications. The plants were inoculated with *B. cinerea* by applying a 5 μl droplet of a suspension of 1 × 10^5^ spores/ml to four leaves per plant. Subsequently the plants were placed under a lid to increase relative humidity to 100% in order to stimulate the infection. 4 and 7 days after *B. cinerea* inoculation (DAI) the disease symptoms were scored by visual inspection. The percentage of leaves in each class was calculated per plant, and disease ratings were assigned on each leaf according to van der Ent et al. (2008). Statistical analysis was done with SPSS Statistics V25 for windows (*χ* ^2^-test; *n* = 10 plants).

## Results

### *lox1* Encodes an Ile-Group Lipoxygenase Whose Expression Is Up-Regulated During the Mycoparasitic Interaction

BLASTp searches in the *T. atroviride* genome database^2^ using *P. anserina* PaLox1 (Pa_2_4370) and PaLox2 (Pa_6_8140) and *A. fumigatus* LoxA (Afu7g00860) as queries for Ile-group lipoxygenases as well as *A. fumigatus* LoxB (Afu4g02770) as query for Val-group lipoxygenases revealed one hit (Triat 33350). In addition, the *T. atroviride* (taxid:452589) database of the NCBI portal^3^ was screened for further potential lipoxygenases by BLASTp analysis with the motifs WRYAK and WL-L/A-K being characteristic for fungal lipoxygenases (Heshof et al., 2014). All potential hits were negated by protein domain prediction with the ExPASy prosite database (de Castro et al., 2006), confirming that the genome of *T. atroviride* comprises only one lipoxygenase-encoding gene. *T. atroviride lox1* codes for a putative iron-containing Ile-group lipoxygenase with a predicted length of 658 amino acids bearing the characteristic WRAK motif. *T. atroviride* Lox1 displays considerable amino acid identity with the already functionally characterized Ile-group Lox proteins of *P. anserina* (PaLox1, 48.8% and PaLox2, 50.9%; Ferrari et al., 2018), *F. oxysporum* (FOXG_04807.2, 39%; Brodhun et al., 2013) and *A. fumigatus* (LoxA, 40.8%; Fischer et al., 2017).

**Figure 1:**
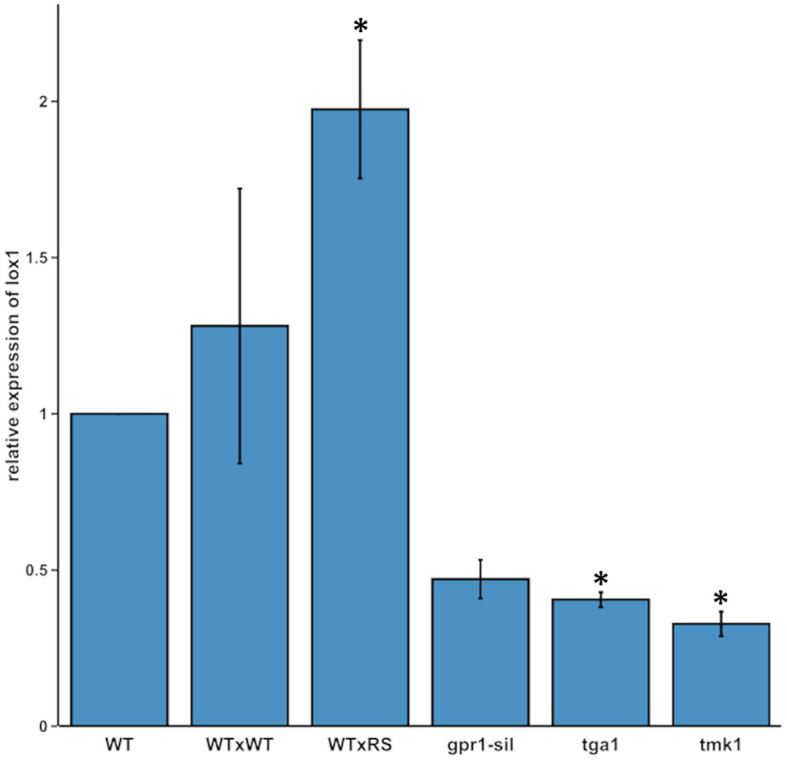
*lox1* gene expression in different *Trichoderma atroviride* strains. Relative transcript ratios of *lox1* in *T. atroviride* wild-type upon axenic growth on PDA (WT), in self-interaction (WTxWT) and in direct confrontation with *Rhizoctonia solani* (WTxRS), as well as in the mutant strains *gpr1*-sil, ∆*tga1*, and ∆*tmk1* grown in axenic culture. *sar1* was used as reference gene and the control sample of the WT [axenic culture on potato dextrose agar (PDA)] served as calibrator, which was arbitrarily assigned the factor 1. The asterisks indicate statistically significant differences compared with the calibrator (*n* = 3; details of statistical evaluation are given in Supplementary Table 3).

Based on previous findings, that 6-PP has antifungal activity and that secondary metabolite production contributes to *Trichoderma* mycoparasitism (Zeilinger et al., 2016), we analyzed *lox1* gene expression in *T. atroviride* upon its mycoparasitic interaction with *R. solani*. We further included mutants interrupted in the mycoparasitism-relevant G-protein and MAP kinase signaling pathways. These were ∆*tga1* (missing the G-α-protein Tga1; Reithner et al., 2005), *gpr1*-sil (silenced in expression of the Gpr1 G-protein coupled receptor-like protein; Omann et al., 2012) and ∆*tmk1* (missing the mitogen-activated-protein kinase Tmk1; Reithner et al., 2007), all of which were previously shown to govern 6-PP production. Transcription of *lox1* significantly increased upon direct contact between *T. atroviride* and *R. solani* compared with the self-confrontation control. In contrast, *lox1* mRNA levels were significantly reduced upon axenic cultivation in all tested mutants compared to the wild-type (Figure 1). While the obtained results on *lox1* expression are consistent with the previously reported, significantly decreased ability of *gpr1*-sil and ∆*tga1* mutants to produce 6-PP (Reithner et al., 2005; Omann et al., 2012), the observed reduced *lox1* expression in the ∆*tmk1* mutant, which has previously been described as a 6-PP over-producer (Reithner et al., 2007), questioned our primary hypothesis of *lox1* being involved in 6-PP biosynthesis.

### Lox1 Is Dispensable for Vegetative Growth and Mycoparasitism

**Figure 2:**
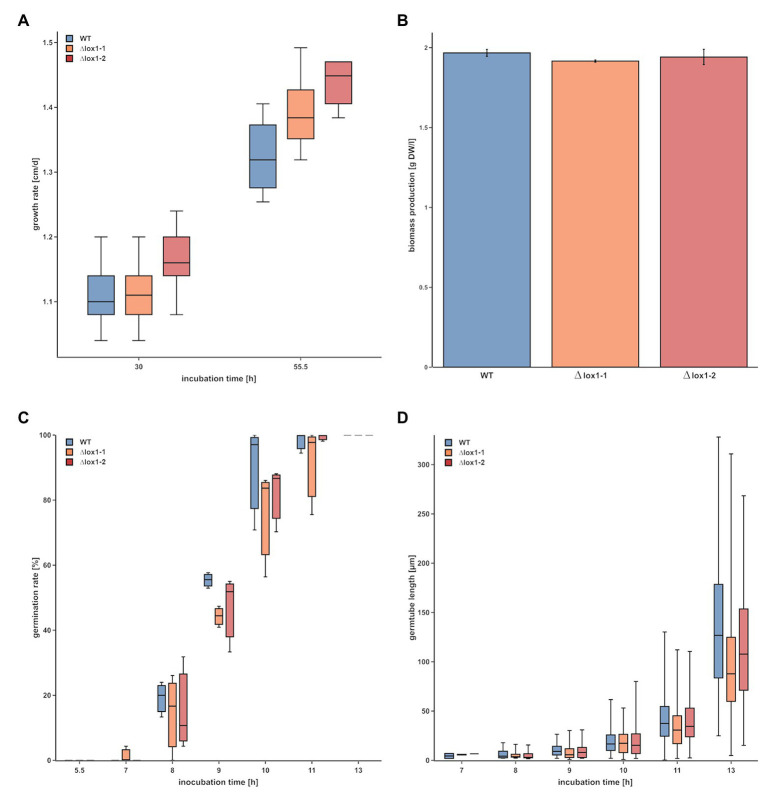
Evaluation of the impact of *lox1* gene deletion on growth and conidial germination of *T. atroviride*. **(A)** Radial growth rate of *T. atroviride* wild-type (WT) and ∆*lox1-1* and ∆*lox1-2* mutants on PDA after cultivation under light-dark conditions for 30 and 55 h. **(B)** Biomass production of *T. atroviride* wild-type (WT) and ∆*lox1-1* and ∆*lox1-2* upon cultivation in potato dextrose broth (PDB) for 50 h. **(C)** Conidial germination rate and **(D)** germ tube development of *T. atroviride* wild-type and ∆*lox1-1* and ∆*lox1-2* after 5.5, 7, 8, 9, 10, 11, and 13 h upon cultivation in PDB. No overall statistically significant differences between the WT and the deletion mutants were found for all four analyses (*n* ≥ 3; details of statistical evaluation are given in Supplementary Table 3).

**Figure 3:**
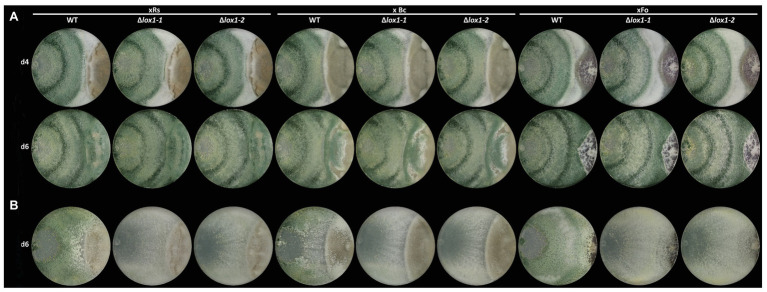
Plate confrontation assays of *T. atroviride* and the *lox1* deletion mutants against different host fungi. The mycoparasitic behavior of *T. atroviride* wild-type (WT) and the lipoxygenase-deficient mutants ∆*lox1-1* and ∆*lox1-2* against *R. solani* (xRs), *Botrytis cinerea* (xBc) and *Fusarium oxysporum* (xFo) was assessed after 4 (d4) and 6 days (d6) of incubation on PDA under light-dark (LD) conditions **(A)** or after 6 days (d6) of incubation in complete darkness (DD; **B**). A representative image of four biological replicates (*n* = 4) is shown.

To obtain clarity about the involvement of *lox1* in 6-PP biosynthesis, we generated *lox1* gene deletion mutants by replacing the *lox1* open reading frame by a hygromycin resistance cassette. Screening of the resulting 120 hygromycin B resistant transformants led to two mitotically stable mutants with a single copy integration of the *lox1* gene deletion construct (Supplementary Figure 2). The ∆*lox1* mutants exhibited a radial growth rate on solid media (PDA; Figure 2A) and a biomass production in liquid culture (PDB; Figure 2B) similar to the wild-type. Accordingly, conidial germination (Figure 2C) and germ tube development (Figure 2D) were unaffected by deletion of *lox1*. To test whether *lox1* affects *T. atroviride* mycoparasitism, plate confrontation assays against *R. solani*, *B. cinerea*, and *F. oxysporum* were performed. After 4 days of incubation, the wild-type and the ∆*lox1* mutants established mycoparasitic interaction with the host fungi. After 6 days, both the wild-type and ∆*lox1* had fully overgrown *R. solani* and *B. cinerea*. In contrast, *F. oxysporum* could solely upon incubation under complete darkness be fully overgrown by the wild-type and the ∆*lox1* mutants after 6 days, but not under light-dark conditions (Figure 3). These results suggest that *lox1* is dispensable for the mycoparasitic attack.

### Lox1 Is Involved in Oxylipin Biosynthesis, but Dispensable for 6-PP Production

**Figure 4:**
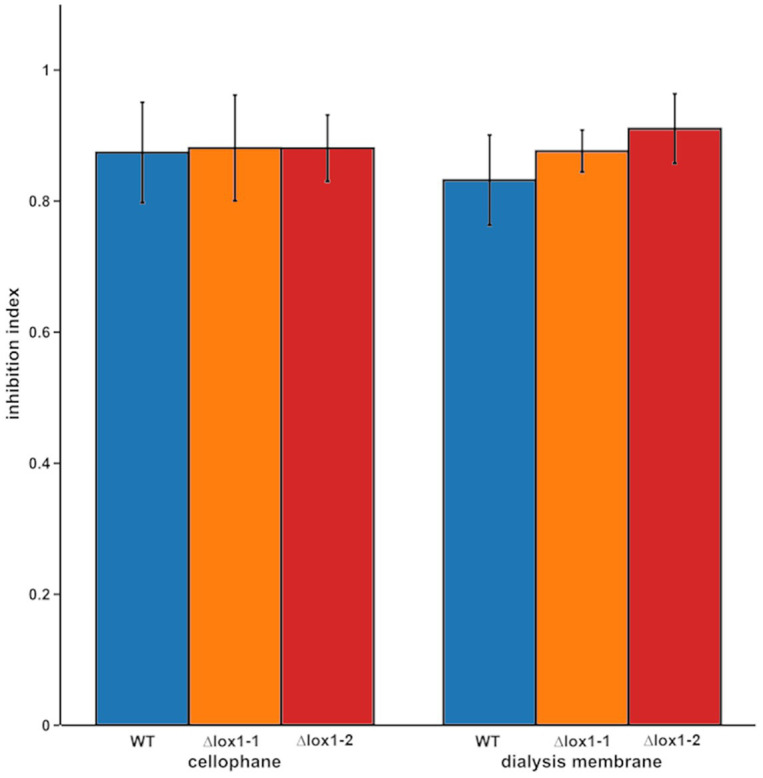
Inhibition of *B. cinerea* by secreted metabolites of *T. atroviride* wild-type and the *lox1* deletion mutants. Indexes of *B. cinerea* growth inhibition by secreted metabolites of *T. atroviride* wild-type (WT) and ∆*lox1-1* and ∆*lox1-2* mutants after cultivation on cellophane or dialysis membranes (MWCO 14 kDa). No statistically significant differences between the wild-type and the deletion mutants, as well as between the respective membrane types were found (*n* = 3; details of statistical evaluation are given in Supplementary Table 3).

**Figure 5:**
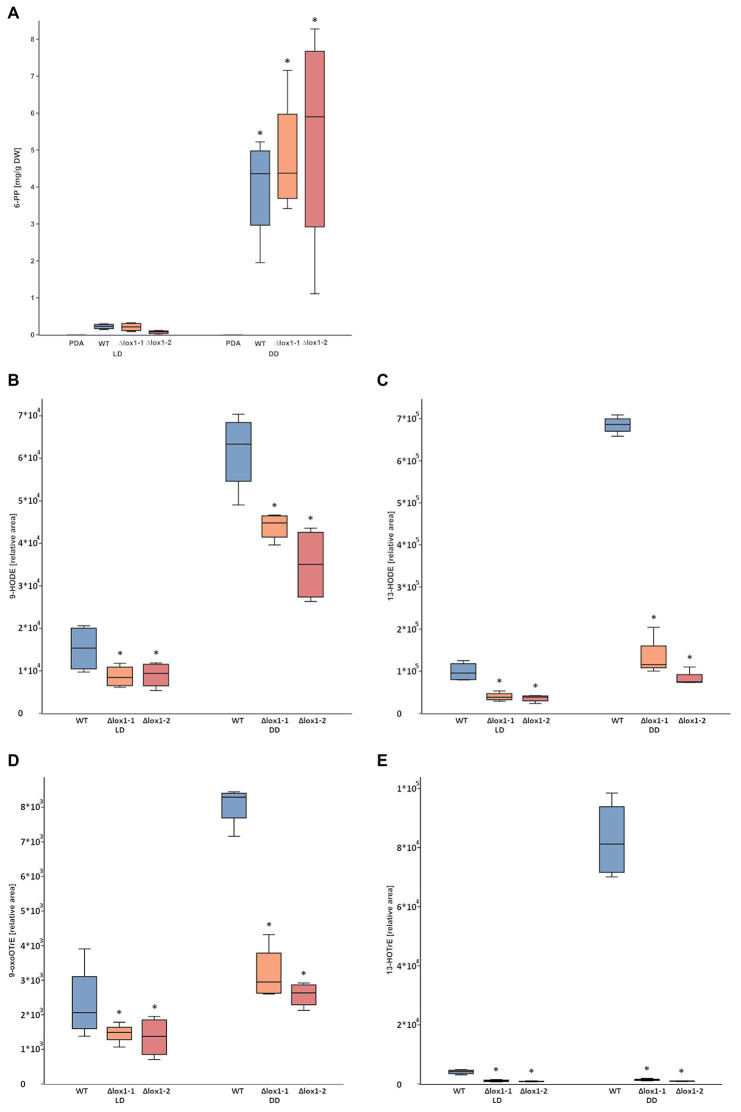
6-pentyl-α-pyrone (6-PP) and oxylipin production by *T. atroviride* wild-type and the *lox1* deletion mutants. **(A)** Amount of 6-PP secreted into the agar (PDA) by *T. atroviride* wild-type (WT) and ∆*lox1-1* and ∆*lox1-2* mutants upon cultivation under light-dark conditions (LD) or in complete darkness (DD). Metabolites were extracted and measured by liquid-chromatography coupled to high-resolution mass spectrometry (LC-HRMS). The results represent values normalized to mycelial dry weight (DW). The asterisks indicate statistically significant differences of DD compared with LD (*n* = 4; details of statistical evaluation are given in Supplementary Table 3). No significant differences were observed between the wild-type and the deletion mutants. **(B–D)** Relative amounts of the oxylipins 9-HODE **(B)**, 13-HODE **(C)**, 9-oxoOTrE **(D)**, and 13-HOTrE **(E)** in *T. atroviride* wild-type (WT) and ∆*lox1-1* and ∆*lox1-2* mutants upon cultivation under light-dark conditions (LD) or in complete darkness (DD) after 5 days of growth. Oxylipins were extracted and measured by liquid-chromatography coupled to high-resolution tandem mass spectrometry with multiple-reaction-monitoring mode (HPLC-MS/MS-MRM). The results represent values normalized to an internal standard. The asterisks indicate statistically significant differences of the ∆*lox1* mutants compared with the WT. Significant differences not indicated here were also observed between LD and DD conditions (*n* = 4; details of statistical evaluation are given in Supplementary Table 3).

Since *T. atroviride* produces several antifungal secondary metabolites including 6-PP, the antifungal activity of the ∆*lox1* mutants was determined. The wild-type and the ∆*lox1* mutants were, independent of the applied membrane type, to a similar extent able to inhibit the spore germination and growth of *B. cinerea* by secreted antifungal substances (inhibition indices of 0.8–0.9; Figure 4). Quantification of 6-PP secreted into the agar by *T. atroviride* cultures grown either in the dark or under light-dark conditions revealed that the wild-type as well as the *lox1* deletion mutants produced significantly higher quantities of this secondary metabolite in darkness than in the presence of light. However, similar amounts of 6-PP were secreted into the agar by the ∆*lox1* mutants and the wild-type under each of the given cultivation conditions (Figure 5A). Our results hence indicate a strongly light-dependent biosynthesis of 6-PP in *T. atroviride* which, however, is not affected by *lox1* gene deletion.

Analysis of the linoleic acid-derived oxylipins 9-HODE (Figure 5B) and 13-HODE (Figure 5C) and the α-linolenic acid-derived oxylipins 9-oxoOTrE (Figure 5D) and 13-HOTrE (Figure 5E) revealed significantly reduced levels in both ∆*lox1* mutants compared to the wild-type in either dark or light-dark grown cultures, indicating a role of Lox1 in the biosynthesis of selected oxylipins.

### Lox1 Is Required for Triggering Injury-Induced Conidiation

Mycelial injury was described to result in the formation of asexual reproduction structures in *T. atroviride* (Steyaert et al., 2010; Hernández-Oñate et al., 2012). Injury-triggered conidiation involves – besides other processes – lipid metabolism and the production of oxylipins and reactive oxygen species (Hernández-Oñate et al., 2012). Interestingly, *lox1* was found among the injury-induced genes with transcripts rapidly accumulating after the damage (Hernández-Oñate et al., 2012). We hence analyzed the impact of *lox1* on conidiation triggered by mechanical injury and blue light.

**Figure 6:**
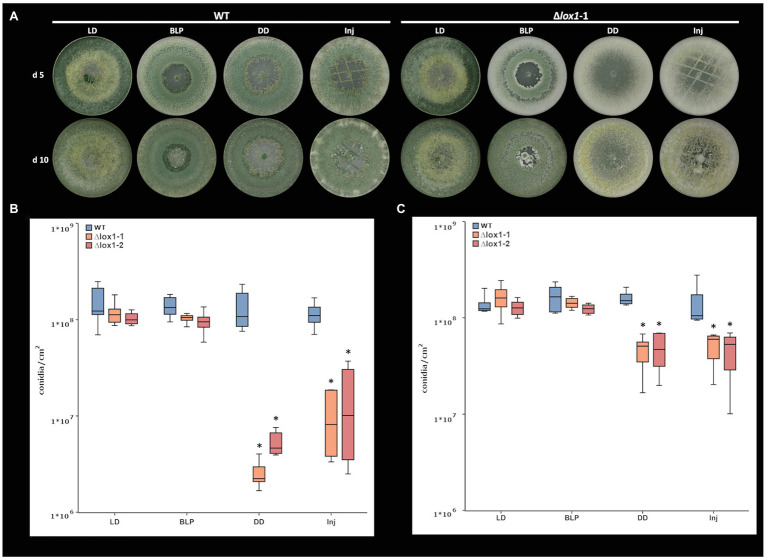
Light‐ and injury-triggered morphotype and conidiation rate of *T. atroviride* wild-type and the *lox1* deletion mutants. Morphotype **(A)** and number of conidia produced by *T. atroviride* wild-type (WT) and ∆*lox1-1* and ∆*lox1-2* mutants after incubation on PDA at 25°C for 5 days (d5; **B**) or 10 days (d10; **C**). Fungi were grown under light-dark conditions (LD) or in complete darkness (DD), or in complete darkness for 48 h and then illuminated for 10 min with a blue-light pulse (BLP) or injured by cutting with a scalpel (Inj). Statistically significant differences were observed between the wild-type and the deletion mutants and upon cultivation in DD and Inj compared to LD and BLP. The asterisks summarize and indicate the significant differences between the cultivation conditions and strains (d5 *n* ≥ 6; d10 *n* ≥ 4; details of statistical evaluation are given in Supplementary Table 3).

The *T. atroviride* wild-type strain P1 responded to mycelial injury and blue light with the production of green conidia. These responses somewhat differed from the previously reported behavior of *T. atroviride* strain IMI 206040 (Casas-Flores et al., 2004; Hernández-Oñate et al., 2012), as conidia formation not exclusively occurred at the wounding sites but all-over the colony, and conidiation intensity in darkness was similar to conidiation upon growth under light-dark conditions (Speckbacher et al., 2020). Compared with the wild-type, loss of *lox1* reduced conidiation in complete darkness from 1.36 × 10^8^ to 3.91 × 10^6^ conidia/cm^2^, which corresponds to a 97% reduction after growth for 5 days, and 1.60 × 10^8^ to 4.74 × 10^7^ conidia/cm^2^, which corresponds to a 70% reduction after growth for 10 days. In addition, conidiation was not triggered by mechanical injury in the deletion mutants which showed an 88% reduced conidiation (1.35 × 10^7^ conidia/cm^2^ in the mutants compared to 1.14 × 10^8^ conidia/cm^2^ in the wild type) after growth for 5 days and a 66% reduction (4.91 × 10^7^ conidia/cm^2^ in the mutants compared to 1.45 × 10^8^ conidia/cm^2^ in the wild type) after growth for 10 days (Figure 6). Contrary, illumination with a blue-light-pulse led to a more or less complete rescue of the conidiation phenotype of ∆*lox1* mutants.

**Figure 7:**
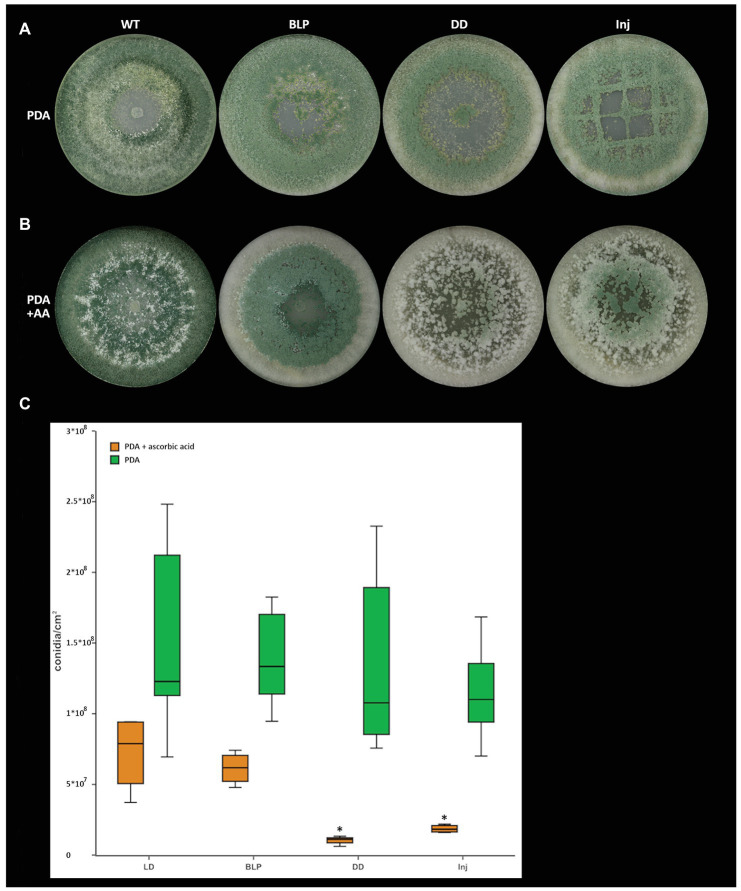
Light‐ and injury-triggered morphotype and conidiation rate of *T. atroviride* wild-type on PDA supplemented with the antioxidant ascorbic acid. Morphotype of *T. atroviride* wild-type grown on **(A)** PDA, on **(B)** PDA in the presence of 4 mg/ml ascorbic acid (AA) and **(C)** number of conidia produced by the wild-type after 5 days of cultivation in the presence of 4 mg/ml ascorbic acid upon growth under light-dark conditions (LD), in complete darkness (DD), or in complete darkness for 48 h followed by illumination for 10 min with a blue-light pulse (BLP) or followed by mechanical injury (Inj). The asterisks indicate and summarize the significant differences between the cultivation conditions (*n* = 4; details of statistical evaluation are given in Supplementary Table 3).

Since lipoxygenases oxygenate their substrates (Oliw, 2002; Heshof et al., 2014), we next wanted to assess if the addition of an antioxidant could provoke a morphotype in the wild-type that is similar to that of ∆*lox1*. Similar as described for sclerotia formation in *R. solani* (Georgiou and Petropoulou, 2001), the addition of ascorbic acid led to a reduction of conidiation by 82% (1.56 × 10^8^ conidia/cm^2^ on PDA compared to 7.23 × 10^7^ conidia/cm^2^ on PDA + ascorbic acid) in the *T. atroviride* wild-type under light-dark conditions. In addition, conidiation was further reduced by 74% (7.23 × 10^7^ conidia/cm^2^ uninjured in LD compared to 1.86 × 10^7^ conidia/cm^2^ injured) upon injury in the presence of ascorbic acid, and by 86% (1.05 × 10^7^ conidia/cm^2^ in DD compared to 7.23 × 10^7^ in LD) upon growth in complete darkness (Figure 7).

### Lox1 Affects the Production of Secondary Metabolites in a Light-Dependent Manner

**Figure 8:**
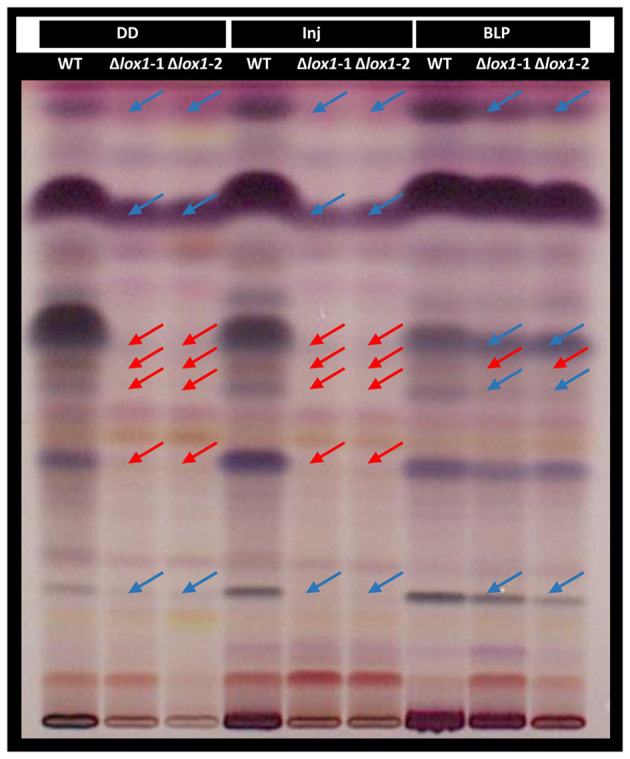
High-performance thin-layer chromatography (HPTLC) analysis of *T. atroviride* wild-type and the *lox1* deletion mutants. *T. atroviride* wild-type (WT) and the lipoxygenase-deficient mutants ∆*lox1-1* and *∆lox1-2* were grown in complete darkness (DD) for 48 h followed by mechanical injury (Inj) or treatment with a blue-light pulse (BLP). Control plates were incubated under complete darkness. Metabolites were extracted from the agar and analyzed by HPTLC. Metabolite patterns were visualized by visible light after derivatization with p-anisaldehyde:sulfuric acid reagent. Major differences in the secondary metabolite patterns between the wild-type (WT) and ∆*lox1-1* and ∆*lox1-2* are highlighted. Red arrows indicate missing bands and blue arrows indicate bands with reduced intensity compared to the WT. A representative image of three biological replicates (*n* = 3) is shown.

Based on our results indicating an effect of Lox1 on light-dependent and injury-induced conidiation and the fact that light affects fungal secondary metabolism (Yu and Keller, 2005; Rodriguez-Romero et al., 2010; Tisch and Schmoll, 2010), we aimed at getting a rough overview on the global secondary metabolite patterns secreted by *T. atroviride* wild-type and ∆*lox1* upon cultivation in complete darkness, mechanical injury and blue-light-pulse treatment. HPTLC analysis of the wild-type samples upon derivatization with p-anisaldehyde:sulfuric acid reagent – allowing the detection of phenols, sugars, steroids, and terpenes (Stahl and Glatz, 1982) – confirmed the production of several compounds of these substance classes by *T. atroviride* as described previously (Mukherjee et al., 2012). Only minor differences in the presence and intensity of individual compounds between the unscathed and injured samples from complete darkness were visible. The metabolite patterns of both ∆*lox1* mutants were highly similar to each other, but clearly differed from the wild-type in the absence of light (Figure 8). Compared with the wild-type, several compounds were missing or had a decreased intensity in the extracts from the unscathed and injured dark-grown mutant cultures. Metabolite patterns of the mutants and the wild-type treated with a blue-light-pulse, however, were more similar to each other, with the blue-light treatment leading to a shift of the mutant’s banding pattern towards that of the wild-type. This indicates that Lox1 activity significantly contributes to secondary metabolite production upon growth in complete darkness.

**Figure 9:**
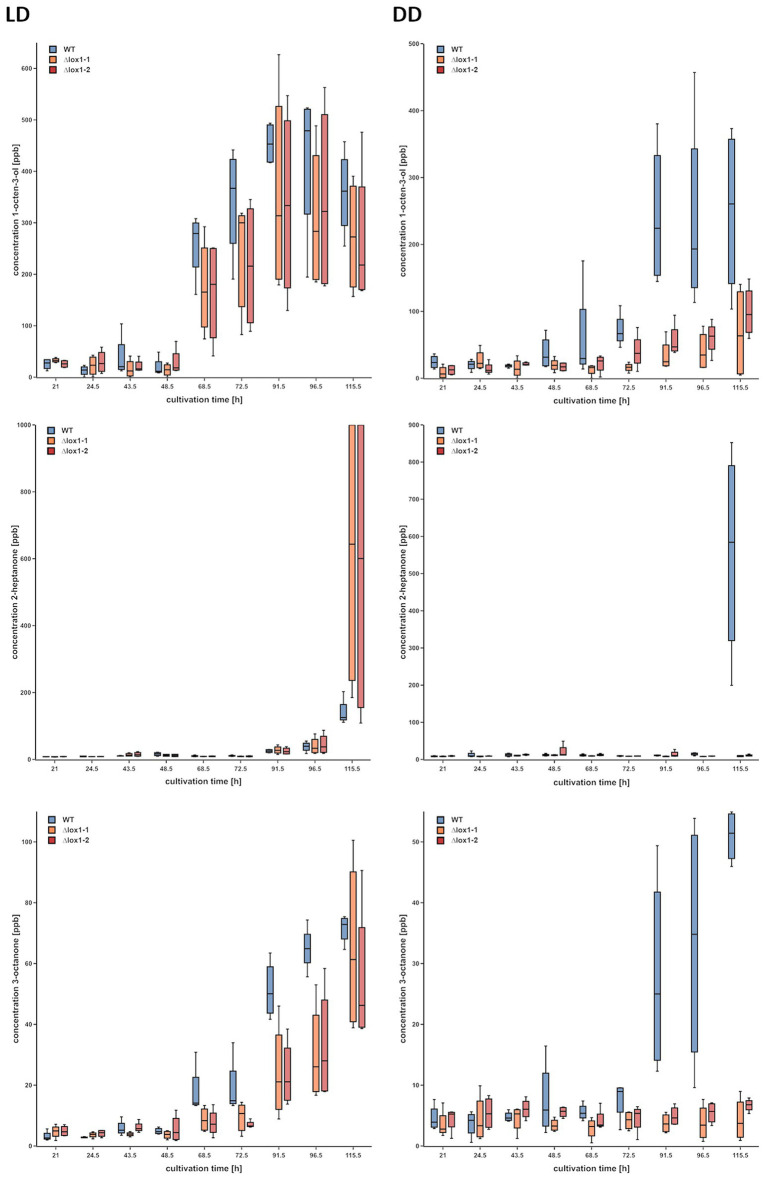
Gas chromatography – ion mobility spectrometry (GC-IMS) headspace analysis of volatile organic compounds (VOCs) emitted by the *T. atroviride* ∆*lox1* deletion mutants in a strongly reduced manner under dark conditions compared to the wild-type. Concentrations (ppb) of 1-octen-3-ol (row 1), 2-heptanone (row 2) and 3-octanone (row 3) in the headspace of *T. atroviride* wild-type (WT) and ∆*lox1-1* and ∆*lox1-2* mutants along a cultivation period of 120 h upon incubation under light-dark conditions (LD) or in complete darkness (DD) are given. Statistically significant differences between the WT and the mutants were observed for all three VOCs upon growth in DD; for 3-octanone also in LD (*n* = 4; details of statistical evaluation are given in Supplementary Table 3). Photographs of WT and ∆*lox1-1* cultures used for VOC analyses are given in Supplementary Figure 3.

Lipoxygenases are well known to be involved in the biosynthesis of VOCs in plants (Heiden et al., 2003), but also were reported to be essential for the production of certain volatiles in *P. anserina* (Ferrari et al., 2018). We therefore assessed the effect of *lox1* deletion on light-dependent VOC biosynthesis in *T. atroviride*. Under light-dark conditions, the ∆*lox1* mutants and the wild-type emitted similar VOC profiles. They increasingly and similarly produced 1-octen-3-ol, 2-heptanone, and 3-octanone under these conditions. In contrast, the concentration of these compounds was significantly reduced upon cultivation in complete darkness in the ∆*lox1* mutants compared to the wild-type (Figure 9).

### Volatile-Mediated Induction of Plant Resistance Is Enhanced Upon *lox1* Gene Deletion

**Figure 10:**
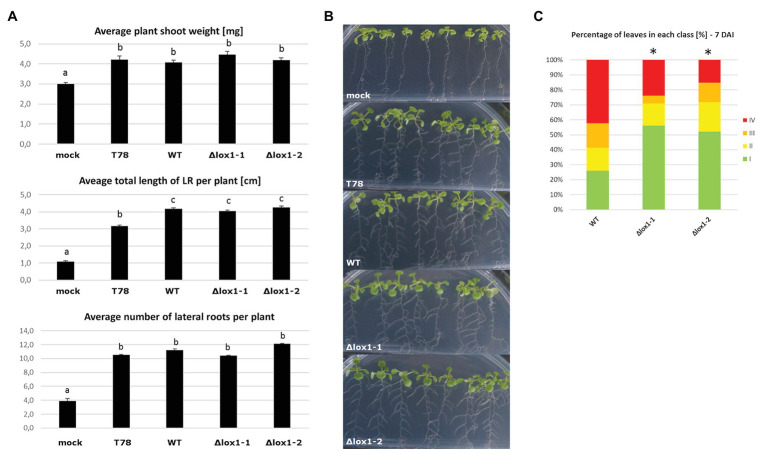
Effect of *T. atroviride* VOCs on *Arabidopsis thaliana* root development and *B. cinerea*-mediated disease. **(A)** Average plant shoot weight, total length of lateral roots per plant and number of lateral roots per plant after *A. thaliana* seedlings were exposed to VOCs of *Trichoderma harzianum* T78 (T78), *T. atroviride* wild-type (WT), or the lipoxygenase-deficient ∆*lox1-1* or ∆*lox1-2* mutants for 6 days in split-plate assays. Results shown are means ± SD. The lowercase letters indicate statistically significant differences compared with mock-treated (mock) control plants (one-way ANOVA; Tukey HSD test; *p* < 0.05; *n* = 3 plates, with 10 plants each). **(B)** Representative photographs of *Arabidopsis* seedlings treated as in **(A)**. **(C)** Quantification of disease symptoms in *Arabidopsis* leaves after inoculation with *B. cinerea*. After 3 days of exposure to VOCs of *T. atroviride* wild-type (WT), or the lipoxygenase-deficient ∆*lox1-1* or ∆*lox1-2* mutants in split-plate assays, seedling were transplanted into pots and infected 3 weeks later with *B. cinerea* spores. Disease severity was scored according to four classes (I – no visible disease symptoms; II – non-spreading lesion; III – spreading lesion without tissue maceration; IV – spreading lesion with tissue maceration and sporulation of the pathogen) 7 days after infection (DAI) and the percentage of leaves in each class was calculated per plant. The asterisks indicate statistically significant differences compared with WT-treated plants (*χ* ^2^ test; *n* = 10 plants). Results and photographs from 4 DAI are given in Supplementary Figure 4. Statistics was done in SPSS Statistics V25 for windows.

VOC mixtures from certain *Trichoderma* species stimulate plant growth and prime the plant’s immune system, even in the absence of physical contact with the plant (Kottb et al., 2015; Martinez-Medina et al., 2016; Martínez-Medina et al., 2017). Since ∆*lox1* mutants differed in VOC production from the wild-type, we wanted to assess the effect of the mutants on growth and induction of systemic resistance in *A. thaliana* plants using a split growth system. *T. harzianum* T-78, a strain known to induce systemic resistance against the leaf pathogen *B. cinerea* (Martínez-Medina et al., 2013), was used as a positive control. *T. atroviride* wild-type and its ∆*lox1* mutants stimulated the growth of *A. thaliana* seedlings in root and shoot similar to *T. harzianum* (Figures 10A,B). In an assay assessing the resistance of *A. thalliana* plants against *B. cinerea* mediated by *Trichoderma*-derived VOCs, plants exposed to VOCs emitted by the ∆*lox1* mutants developed significantly less-severe disease symptoms compared to plants treated with VOCs of the *T. atroviride* wild-type (Figure 10C).

## Discussion

Isotopic labeling experiments in *T. atroviride* in the early 1990s suggested the oxidation of linoleic acid by a lipoxygenase as a first step in the biosynthesis of 6-PP, one of *T. atroviride*’s main secondary metabolites with antifungal and plant growth promoting properties (Scarselletti and Faull, 1994; Vinale et al., 2008; Garnica-Vergara et al., 2016; Ismaiel and Ali, 2017). In this study, we showed, however, that 6-PP biosynthesis in *T. atroviride* is not affected by deletion of the single lipoxygenase-encoding gene *lox1* encoded in its genome. Interestingly though, our results revealed that 6-PP biosynthesis was repressed in the presence of light, while substantial amounts of this secondary metabolite were secreted upon growth in complete darkness. Similarly, oxylipin production in *T. atroviride* turned out to be influenced by light and by *lox1* gene deletion. In *Aspergillus nidulans* and other filamentous ascomycetes, the velvet complex coordinates the light signal with fungal development and secondary metabolism. The fully functional velvet complex consisting of LaeA, VelB, and VeA only forms under dark conditions, thereby controlling the epigenetic activity of LaeA, which subsequently regulates the expression of secondary metabolite gene clusters (Bayram et al., 2008). Diverse compounds based on the 2*H*-pyran-2-one structure are known to be produced by filamentous fungi, including *Aspergillus*, *Penicillium*, *Fusarium*, and *Trichoderma* species (Dickinson, 1993). Although the 2*H*-pyran-2-one metabolite fusalanipyrone from *Fusarium solani* has been suggested to be of monoterpenoid origin (Abraham and Arfmann, 1988), a recent study demonstrated that its stereoisomer gibepyrone A from *Fusarium fujikuroi* has a polyketide nature (Janevska et al., 2016). It is now widely accepted that α-pyrones are predominantly synthesized *via* the polyketide pathway, which has much in common with fatty acid biosynthesis such as the use of simple building blocks (e.g., acetyl-CoA and malonyl-CoA) and the mechanism of chain elongation (Schäberle, 2016). Accordingly, we suggest that 6-PP biosynthesis in *T. atroviride* most probably involves one of the 18 polyketide synthases encoded in its genome (Schmoll et al., 2016).

To date little is known on the distinct physiological roles of fungal lipoxygenases. Deletion of the Val-group lipoxygenase *Aflox* in *A. flavus* led to a major reduction in cell-density dependence of sclerotia and conidia formation (Horowitz Brown et al., 2008). In *A. fumigatus* deletion of *loxB*, a Val-group lipoxygenase-encoding homologue of *Aflox*, delayed swelling as well as germination of conidia in the presence of arachidonic acid (Fischer et al., 2017). In this study, we identified *T. atroviride* Lox1 as an Ile-group lipoxygenase bearing the typical WRAK motif. Interestingly, *lox1* gene deletion resulted in a strongly light-counteracted phenotype: All observed morphologic and metabolic alterations of the ∆*lox1* mutants were apparent only upon growth in complete darkness, but rescued by blue-light treatment as well as circadian white-light exposure. We speculate that for instance enzyme-independent lipid peroxidation driven by enhanced levels of reactive oxygen species generated in the presence of light could mask the effect of *lox1* gene deletion. We obtained evidence for this assumption from antioxidant treatment of the wild-type which mimicked the ∆*lox1* conidiation phenotype. Loss of *lox1* led to a massively reduced production of conidia upon incubation in complete darkness. Furthermore, ∆*lox1* mutants lost the ability to produce certain secondary metabolites when grown in complete darkness, as evidenced by the obtained HPTLC pattern and by VOC analysis. Interestingly, mechanical injury of ∆*lox1* mycelia could not induce conidiation or recover metabolite biosynthesis. A similar phenotype has been reported for *T. atroviride* Δ*lae1* mutants missing the methyltransferase Lae1/LaeA, a global regulator that affects the expression of secondary metabolite gene clusters and controls sexual and asexual development in many ascomycetes (Sarikaya-Bayram et al., 2015). Similar to Δ*lox1*, Δ*lae1* strains did neither sporulate in darkness nor upon mechanical injury and interestingly, *lox1* transcript levels were strongly down-regulated in Δ*lae1* (Karimi Aghcheh et al., 2013).

Our results suggest that injury-triggered asexual reproduction in *T. atroviride*, which, among others, involves lipid-signaling pathways (Steyaert et al., 2010; Hernández-Oñate et al., 2012; Hernández-Oñate and Herrera-Estrella, 2015) is dependent on Lox1 and may involve a regulatory crosstalk between Lae1 and Lox1. This indicates an involvement of Lox1 in an enhanced wound healing process in *T. atroviride*, since conidiation is a major sign for stress response. In accordance with our results, various plant and animal lipoxygenases play an important role in injury and inflammatory responses as well as in wound healing (Bell et al., 1995; Royo et al., 1999; Porta and Rocha-Sosa, 2002; Gronert et al., 2005; Gronert, 2008; Brogliato et al., 2014; Mashima and Okuyama, 2015; Guimarães et al., 2018).

A recent study in *P. anserina* demonstrated a reduced to diminished production of certain VOCs and a strongly decreased ability to repel the nematode *C. elegans* in double-deletion mutants lacking the genes for the two Ile-group lipoxygenases encoded in the *P. anserina* genome (Ferrari et al., 2018). We could not see any effect of *lox1* deletion on the strength and progress of the mycoparasitic attack, irrespective of the applied light regime, in our study. However, *lox1* deletion led to altered VOC production with a largely reduced 1-octen-3-ol, 2-heptanone, and 3-octanone emission upon growth of *T. atroviride* in complete darkness. These results are in accordance with previous studies indicating that the oxygenation of linoleic-, linolenic-, or arachidonic-acid is the initial step in oxylipin biosynthesis, followed by additional steps that finally lead to a variety of oxylipins such as C8 volatiles (Kuribayashi et al., 2002; Brodhun and Feussner, 2011; Karrer and Rühl, 2019). Altered VOC emission profiles were also described for *P. anserina Lox^∆^* mutants which, however, still produced three C8 molecules including 1-octen-3-ol and 3-octanone, whose synthesis was hence suggested by the authors to rely on cyclooxygenase enzymes (Ferrari et al., 2018). Screening of the *T. atroviride* genome revealed at least two cyclooxygenases with high sequence similarity to *P. anserina* PaCox1 and PaCox2, which could be responsible for 1-octen-3-ol and 3-octanone biosynthesis in the presence of light in *T. atroviride*. Accordingly, all identified VOCs that differed between the ∆*lox1* mutants and the wild-type were oxygenated. This indicates that their biosynthesis, at least partially, relies on lipoxygenases or cyclooxygenases. The reported roles of those volatiles are diverse (Combet et al., 2006; Gupta et al., 2014). 1-octen-3-ol for instance is responsible for the characteristic mushroom-smell and is produced by a broad variety of fungi. It is known to attract insects (Kline et al., 2007), exhibit antibacterial and slight antifungal properties (Xiong et al., 2017) and inhibit the germination of plant seeds and the growth of seedlings (Hung et al., 2014). Although the effect of VOCs emitted by the ∆*lox1* mutants and the wild-type on *Arabidopsis* root architecture and shoot weight was similar in our study, the mutants had an enhanced ability to induce systemic resistance against *B. cinerea*. This suggests a role of Lox1 in the biosynthesis of volatiles negatively affecting the elicitation of induction of systemic resistance in *Arabidopsis*.

In summary, we characterized the Ile-group lipoxygenase Lox1 in the mycoparasitic fungus *T. atroviride*. We demonstrate that Lox1 affects the response of *T. atroviride* to light and injury and is involved in conidiation and the biosynthesis of secondary metabolites – including the VOCs 1-octen-3-ol, 2-heptanone, and 3-octanone – under dark growth conditions. These data provide novel insights into the light-dependent role of fungal lipoxygenases.

## Data Availability Statement

All datasets generated for this study are included in the article/Supplementary Material.

## Author Contributions

VS and SZ conceived and directed this study and drafted the manuscript. VS contributed to all parts of analysis. VR conducted GC-MS and GC-IMS analysis. AM-M conceived and conducted the plant experiments. MD and RS contributed to 6-PP analysis. US contributed to RT-PCR and RT-qPCR analysis. WH, SB, and MS contributed to HPTLC analysis. MB and MR performed oxylipin analysis. All authors contributed to the article and approved the submitted version.

### Conflict of Interest

The authors declare that the research was conducted in the absence of any commercial or financial relationships that could be construed as a potential conflict of interest.

## Footnotes

^1^https://mycocosm.jgi.doe.gov/cgi-bin/dispGeneModel?db=Triat2&id=33350

^2^https://genome.jgi.doe.gov/Triat2/Triat2.home.html

^3^https://blast.ncbi.nlm.nih.gov/Blast.cgi
