# Supplementary material for: The Lipoxygenase Lox1 Is Involved in Light‐ and Injury-Response, Conidiation, and Volatile Organic Compound Biosynthesis in the Mycoparasitic Fungus Trichoderma atroviride
Source: Front Microbiol. 2020 Aug 27;11:2004. doi: 10.3389/fmicb.2020.02004 (PMC7482316; doi:10.3389/fmicb.2020.02004)
Supplement: Supplementary file 1 [file Data_Sheet_1.docx]

Supplementary Material

| primer name | 5´-3´ sequence |
| --- | --- |
| **33350-5KO-F1** | GTAACGCCAGGGTTTTCCCAGTCACGACGGGATTGGCCCAAATGTGGTG |
| **33350-5KO-R1** | TCGGGATCCACTCGTCACACTGGCTCTCGCGGTCTTCTTTGGAGGCTCA |
| **33350-3KO-F1** | ATAGTACCCTCGAGGCAACACAACACTCAGCGAAGGGGGACGATGATTT |
| **33350-3KO-R1** | GCGGATAACAATTTCACACAGGAAACAGCACGCACGTAGTCGAAGTGTT |
| **33350-C-F1** | GAAGAGACGGAGAAGGAAA |
| **33350-C-R1** | CAACGGAGAGCCTTCCCATT |
| **hph-C-R1-right** | TGGGGTGGCGAGTATGTA |
| **33350-RT-F1** | CTGCCAGCATCCTTGGAAGA |
| **33350-RT-R1** | GCCTTCTTACCGAAGCCACT |
| **hph-F2** | ATTGCCGTCAACCAAGCTCT |
| **hph-R2** | CTTCGATGTAGGAGGGCGTG |

**Supplementary Table 1:** Primers applied in this study

| **compound name** | **precursor ion** | **prod ion** | **polarity** | **CE (eV)** | **fragmentor (V)** |
| --- | --- | --- | --- | --- | --- |
| 9-HODEd4 | 299.2 | 172.2 | [M-H]^-^ | 20 | 140 |
| 13-HODE | 295.2 | 195.2 | [M-H]^-^ | 20 | 140 |
| 9-HODE | 295.2 | 171.2 | [M-H]^-^ | 25 | 140 |
| 13-HOTrE | 293.2 | 223.2 | [M-H]^-^ | 18 | 140 |
| 13-HOTrE | 293.2 | 195.2 | [M-H]^-^ | 18 | 140 |
| 9-oxoOTrE | 291.4 | 185.2 | [M-H]^-^ | 18 | 140 |

**Supplementary Table 2:** MRM conditions to detect selected oxylipins


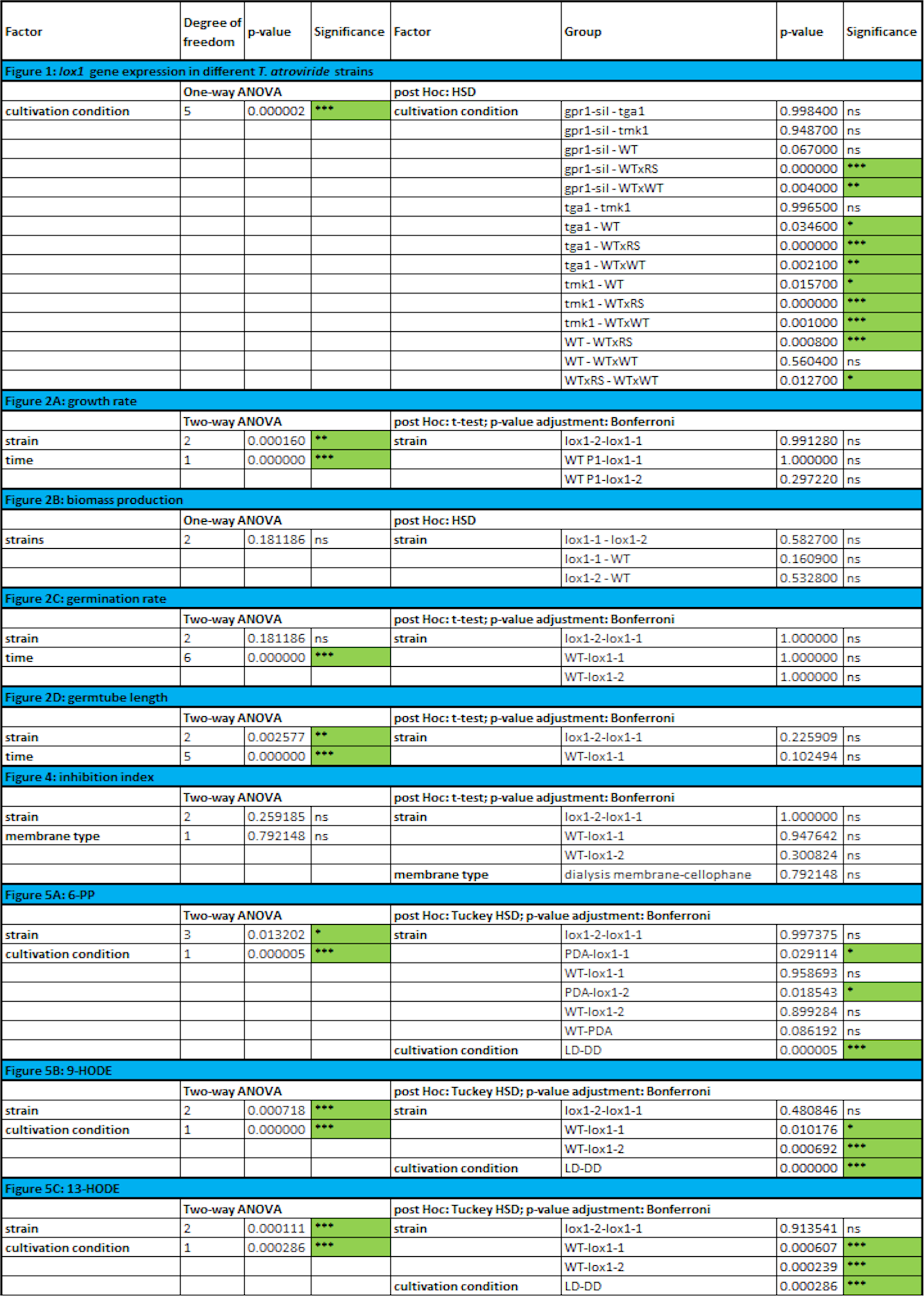


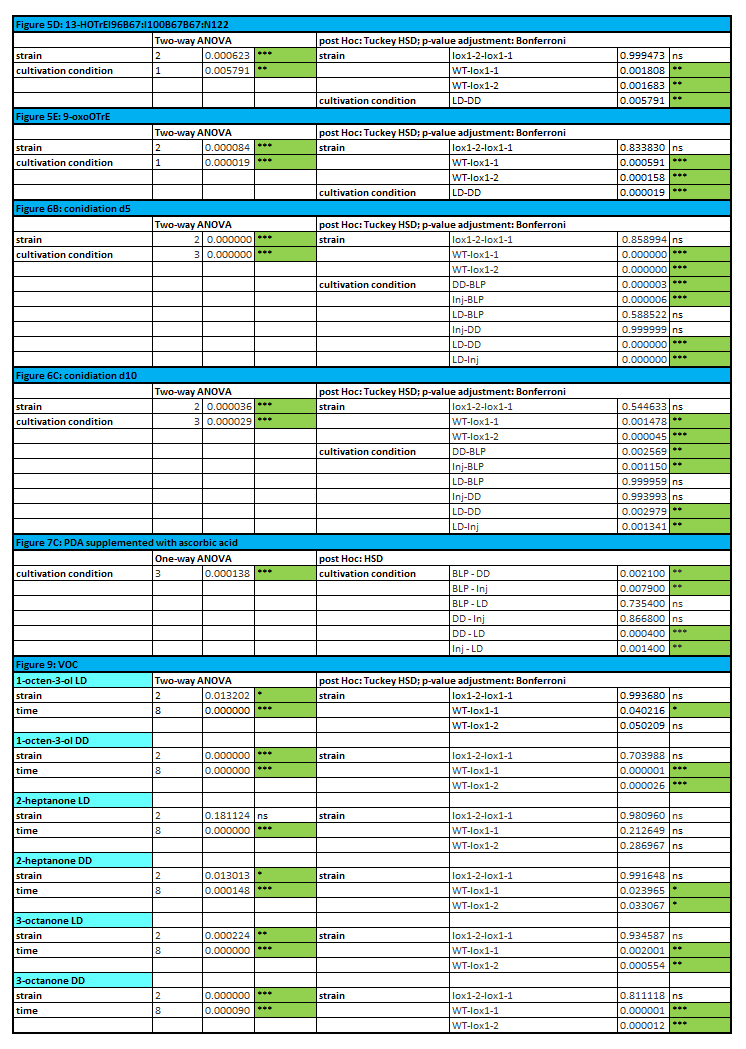


# Supplementary Table 3: Statistical analysis of data given in the figures


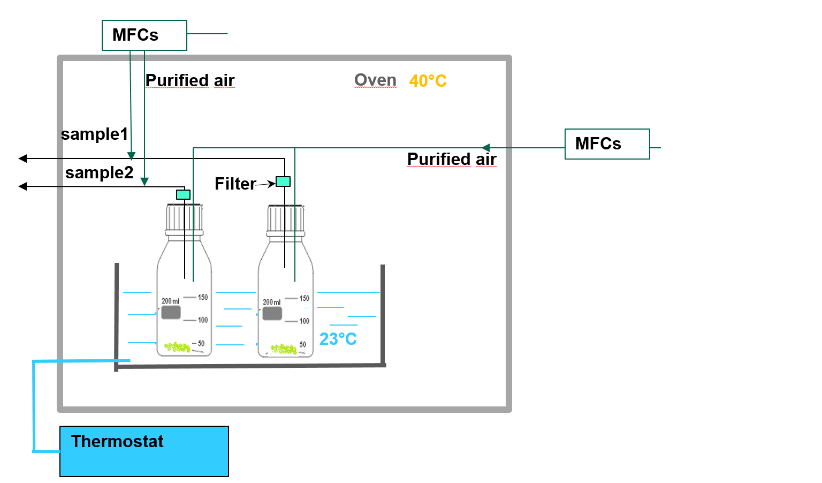


**Supplementary Figure 1:** Set-up of incubator adapted for headspace measurements

**
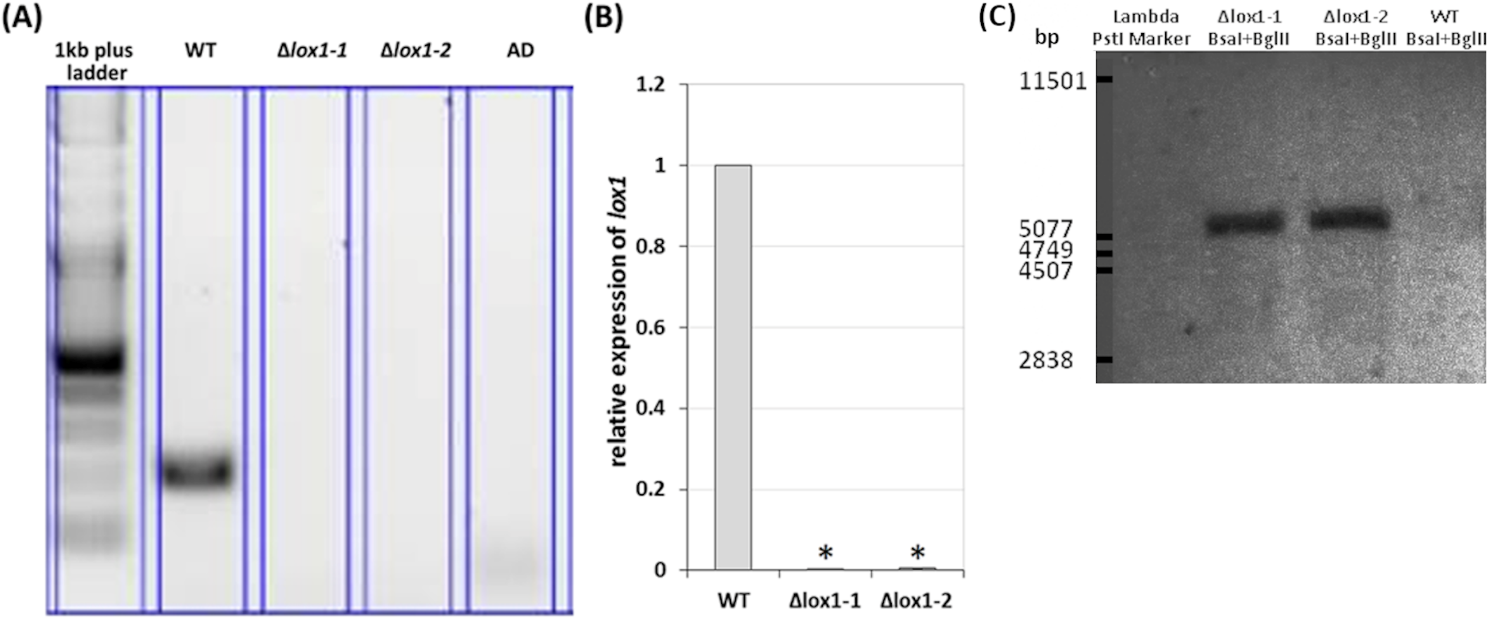
**

**Supplementary Figure 2: Verification of *lox1 gene* deletion. (A)** RT-PCR analysis of *lox1* gene transcription in *T. atroviride* WT, ∆*lox1-1*, ∆*lox1-2* and the no-template-control (AD) upon cultivation on PDA. **(B)** RT-qPCR analysis of *lox1* gene transcription in *T. atroviride* WT and in the *lox1* deletion mutants upon cultivation on PDA. *Sar1* was used as reference gene and the control sample of the WT (axenic culture on PDA) served as calibrator, which was arbitrarily assigned the factor 1. Results shown are means ± SD. The asterisks indicate statistically significant differences compared with the calibrator (paired T-test; p≥0.05; n=3). **(C)** Southern blot analysis to confirm correct, single-copy integration of the *lox1* gene deletion construct. Restriction digest of genomic DNA of ∆*lox1-1* and ∆*lox1-2* with *Bsa*I and *Bgl*II and hybridization with a probe binding within the *hph* gene yielded the expected 5797 bp band spanning both homologous flanks of the deletion cassette.


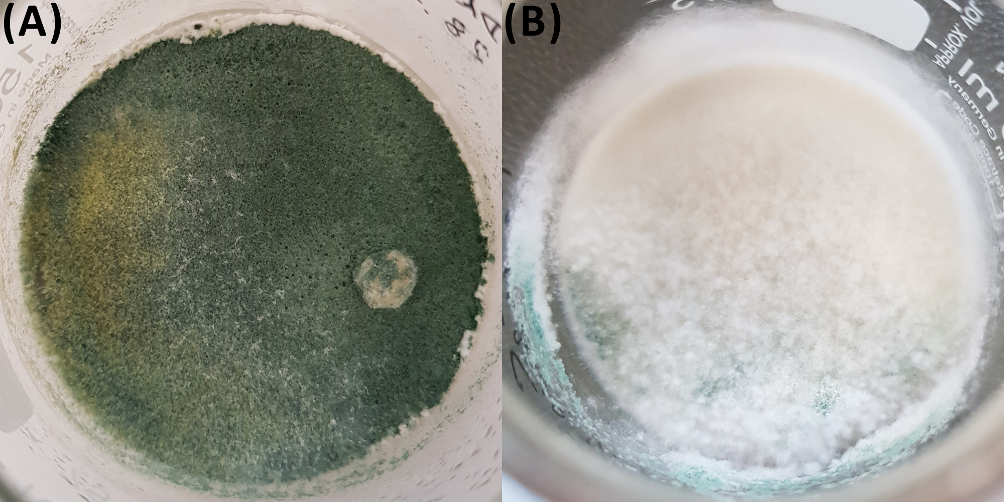


**Supplementary Figure 3: Morphology of cultures used for VOC analyses.** Photos of *T. atroviride* WT **(A)** and ∆*lox1-1* **(B)** cultures after incubation on PDA at 25°C for 5 days under DD conditions in glass bottles covered with aluminium foil.


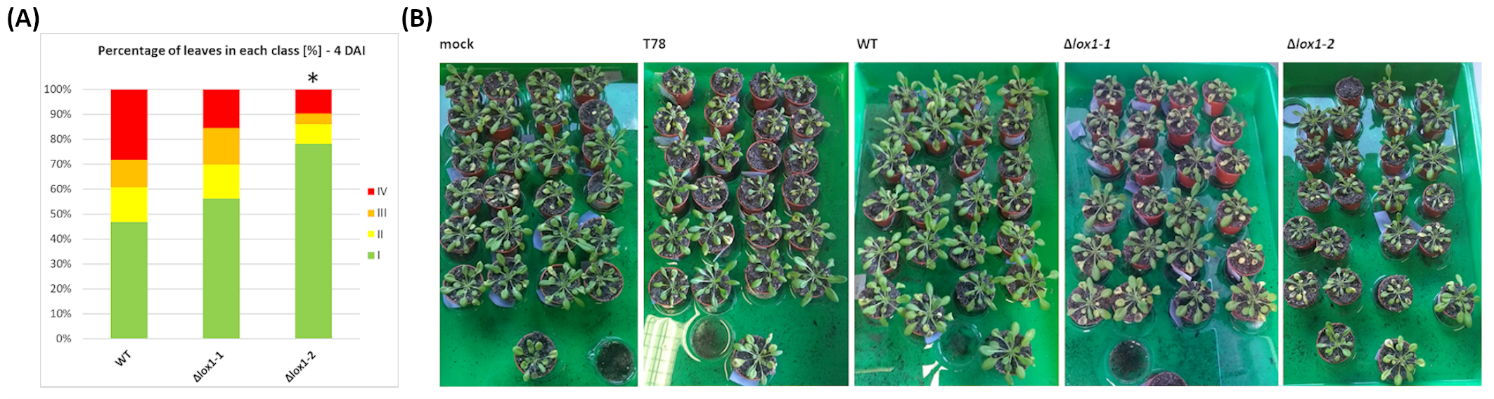


**Supplementary Figure 4: Effect of *T. atroviride* VOCs on *B. cinerea*-mediated disease in *A. thaliana.* (A)** Quantification of disease symptoms in *Arabidopsis* leaves after inoculation with *B. cinerea*. After 3 days of exposure to VOCs of *T. atroviride* wild-type (WT) or ∆*lox1-1* or ∆*lox1-2* mutants in split-plate assays, seedling were transplanted into pots and infected 3 weeks later with *B. cinerea* spores. Disease severity was scored according to four classes (I – no visible disease symptoms; II – non-spreading lesion; III – spreading lesion without tissue maceration; IV – spreading lesion with tissue maceration and sporulation of the pathogen) 4 days after infection (DAI) and the percentage of leaves in each class was calculated per plant. The asterisks indicate statistically significant differences compared with WT treated plants (χ^2^test; n=10 plants). **(B)** Photographs of *B. cinerea* infected *A. thaliana* plants exposed to VOCs of *T. harzianum* T78 (T78), *T. atroviride* wild-type (WT), or ∆*lox1-1* or ∆*lox1-2* mutants 4 DAI with *B. cinerea*.
